# Etiologies des hypertensions artérielles endocrines: à propos d'une série de cas

**DOI:** 10.11604/pamj.2016.23.170.8968

**Published:** 2016-04-07

**Authors:** Naima Bouznad, Ghizlane El Mghari, Nawal El Ansari

**Affiliations:** 1 Service d'Endocrinologie, Diabétologie, Maladies Métaboliques et Nutrition, Laboratoire PCIM/FMPM, CHU Mohamed VI, Marrakech, Maroc

**Keywords:** HTA endocrine, phéochromocytome, hypercorticisme, hyper-aldosteronisme primaire, acromégalie, Endocrine HTA, pheochromocytoma, hypercorticism, primary aldosteronism, acromegaly

## Abstract

Les hypertensions artérielles (HTA) d'origine endocrine restent une cause rare d'HTA, sa prévalence globale n'excède pas 4% des hypertendus. L'intérêt de la recherche des HTA endocrines réside dans la gravité de certaines formes parfois mortelles et le caractère potentiellement curable et réversible de ces HTA. Le but du travail est de déterminer le profil clinique, para clinique, étiologique et thérapeutique des HTA secondaires d'origine endocrine chez les patients suivis au service d'endocrinologie au CHU Mohamed IV à Marrakech. Il s'agit d'une étude descriptive prospective s’étalant sur une période de 4 ans incluant 45 patients ayant une HTA endocrinienne. La moyenne d’âge est de 44,89 ans, avec une nette prédominance du sexe féminine (sexe ratio de 0,49). Les étiologies des HTA endocrines étaient dominées par le phéochromocytome (17 cas), l'hypercorticisme (11 cas) et l'acromégalie (8 cas). L'HTA était paroxystique dans 24,4%. Elle était d'emblée sevère classée grade 3 dans 40% des cas. L'HTA a été compliquée de cardiopathie dans 24% des cas et de néphropathie dans 20% des cas. Le traitement curatif a permis une guérison de l'HTA chez 60% (27 cas). Le diagnostic des HTA secondaires endocrines est parfois difficile du fait de l'absence de spécificité clinique. Il n'est pas exceptionnel que l'HTA soit l'unique manifestation de la maladie. Dans notre travail nous notons le caractère paroxystique et sévère de l'HTA. Le caractère éventuellement curable des HTA endocrines, dans plus des deux tiers des cas, fait qu'il est important de la dépister devant toute HTA sévère, résistante au traitement, ou en présence de signes cliniques, biologiques ou radiologiques évocateurs.

## Abstract (en)

Arterial hypertensions (HTA) of endocrine origin are a rare cause of hypertension; HTA overall prevalence don't exceed 4% of hypertensive patients. Research interest in endocrine HTA is due to the severity of some life-threatening, potentially curable and reversible forms of HTA. The aim of our study was to determine the clinical, paraclinical, etiological and therapeutic profile of secondary HTA of endocrine origin in patients treated in endocrinology department at the University Hospital Mohamed VI in Marrakech. We conducted a prospective, descriptive study spanned 4 years, enrolling 45 patients with endocrine HTA. The average age was 44.89 years, with a clear predominance of women (sex ratio 0.49). Etiology of endocrine HTA was dominated by pheochromocytoma (17 cases), hypercorticism (11 cases) and acromegaly (8 cases). HTA were paroxysmal in 24.4%. HTA were immediately classified as grade 3 severe in 40% of cases. HTA were complicated by heart disease in 24% of cases and by renal disease in 20% of cases. Curative treatment cleared up HTA in 60% of cases (27 cases). The diagnosis of secondary endocrine HTA is sometimes difficult because of the lack of clinical specificity. It is not unusual for HTA to be the only manifestation of the disease. In our study we noted the paroxysmal and severe nature of HTA. The potentially curable nature of HTA in more than two thirds of cases, demostrates the importance of early diagnosis of each severe HTA resistant to treatment or in the presence of suggestive clinical, biological or radiological signs.

## Introduction

L'hypertension artérielle (HTA) est une pathologie très fréquente. Elle touche 972 millions de personnes dans le monde [1, 2]. On distingue 2 entités différentes: les HTA essentielles où aucune cause n'est identifiée (90% des cas) et les HTA secondaires qui sont moins fréquentes, représentent 9,1% des cas [1] et qui peuvent être surtout d'origine rénale ou endocrine [3]. La prévalence des HTA secondaires d'origine endocrine n'est connue qu’à partir de séries hospitalières et sa prévalence globale n′excède pas 4% des hypertendus [4]. Les HTA endocrines sont les causes les plus fréquentes d'HTA secondaire [5]. Dans certains cas, l'HTA n'est pas la manifestation unique ou principale de l'endocrinopathie: c'est le cas des dysthyroïdies, de l'acromégalie, de l'hyperparathyroïdie et du syndrome de Cushing. Par contre, l'HTA est la manifestation cardinale de pathologies endocrines comme l'hyperaldosteronisme primaire (HAP) et le phéochromocytome [4]. Les hypercorticismes et les phéochromocytomes sont des maladies qui nécessitent une prise en charge multidisciplinaire dans des centres de référence [6]. Ces pathologies posent de nombreuses difficultés diagnostiques et thérapeutiques, cependant l'intérêt d'identifier une HTA endocrine réside dans la possibilité de guérison, qui n'existe pas dans le domaine de l'HTA essentielle [1, 7]. Le but de cette étude est de déterminer le profil clinique, para clinique, étiologique et thérapeutique des hypertensions artérielles secondaires d'origine endocrine chez les patients hospitalisés ou consultants au service d'endocrinologie au CHU Mohamed IV.

## Méthodes

Il s'agit d'une étude descriptive prospective, s’étendait sur une période de 4 ans allant du mois de Janvier 2011 au mois de Janvier 2014, chez les patients ayant une HTA endocrine hospitalisés ou suivis en consultation au service d'endocrinologie au CHU Mohammed VI de Marrakech. Les données sont recueillies à partir du dossier médical, et mentionnées sur une fiche d'exploitation préétablie. L'analyse statistique a été effectuée à La saisie et l'analyse des données ont été réalisées à l'aide du logiciel SPSS version 18.

## Résultats

### Descriptif de l’échantillon dans son ensemble (n = 45)

*Caractéristiques socioéconomiques et morphologiques:* La moyenne d’âge des patients était de 44,89 ± 13,82 ans, avec des extrêmes de 17 à 75 ans, et un écart-type de 13,82 ans. On note une nette prédominance du sexe féminin (67%) avec un sexe ratio de 0,49.

### Caractéristiques de l'HTA

*Ancienneté de l'HTA:* 28 patients (60)% présentaient une HTA depuis moins de 1 an; 15 patients (33%) avaient une HTA depuis 1-5 an; 3 patients (7%) avaient une HTA depuis plus de 5 ans.

**Figure 1:**
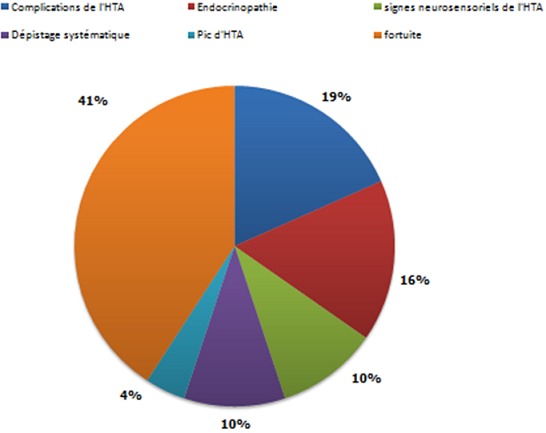
Mode de révélation de l'HTA

**Circonstances de découverte de l'HTA:** les différents motifs du bilan de l'HTA sont représentés dans la Figure 1

**Grade de l'HTA:** on observe chez les 45 patients que: 16 patients (36%) ont une HTA grade I; 11 patients (24%) ont une HTA grade II; 18 patients (40%) ont une HTA grade III.

**Profil de l'HTA:** 29 patients ont une HTA permanente; 11 patients ont une HTA paroxystique; 5 patients ont une HTA permanente avec paroxysme.

**Le profil biologique des patients**: les caractéristiques biologiques des patients sont résumés dans leTableau 1

**Tableau 1:** Profil biologique des patients

Variables	Moyennes ± DS
Kaliémie (mmol/l)	4,47 ± 0,99
Créatininémie (µmol/l)	7,45 ± 1,65
Clairance créatininémie (MDRD)	83 ± 25
Glycémie (mg/dl)	1, 39 ± 0,65
Protéinurie (g/j)	0.15 ± 0.95
Cholestérol total (g/l)	1,72 ± 0.85
C-HDL (g/l)	0,45 ± 0,09
C-LDL (g/l)	1,17 ± 0,55
Triglycérides (g/l)	1,08 ± 0,38

### Les caractéristiques de l'HTA en fonction de l'endocrinopathie

**Figure 2:**
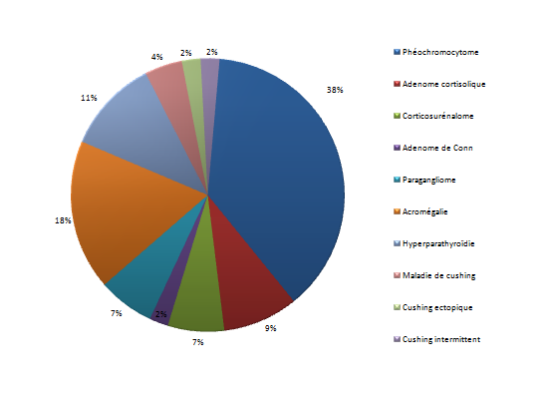
Les étiologies des HTA endocrines

**Les étiologies de l'HTA:** (Figure 2) les étiologies des HTA endocrines sont dominé par: le phéochromocytome chez 17 patients; l'hypercorticisme dans 11 cas; trois cas de paragangliomes; l'acromégalie chez 8 patients; l'adénome de Conn dans 1 seul cas; une hyperparathyroïdie primaire a été notée chez 5 patients.

**Figure 3:**
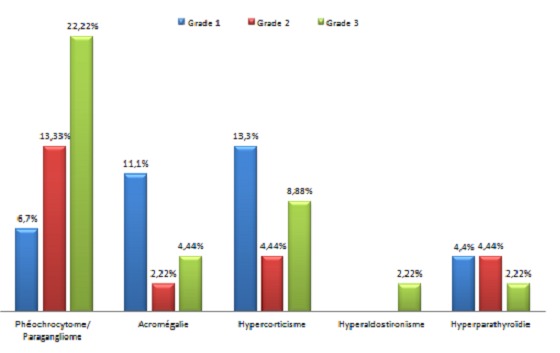
Grade de l'HTA en fonction de l’étiologie

**Grade de l'HTA en fonction de l’étiologie:** chez les patients suivis pour phéochromocytome ou paragangliome, 10 patients ont une HTA Grade 3 (Figure 3).

### Retentissement de l'HTA: (Figure 4)

**Retentissement neurologique:** 3 patients ont un antécédent d'accident vasculaire cérébral (AVC).

**Figure 4:**
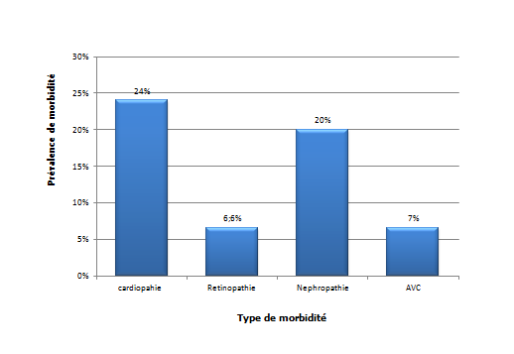
Retentissement de l'HTA endocrine

**Retentissement oculaire**: 3 patients ont une rétinopathie hypertensive au FO

**Retentissement cardiaque**: 3 patients ont des antécédents d'angor; 11 patients ont un retentissement cardiaque; 6 patients ont un HVG; 2 patients ont une cardiomyopathie dilatée; 1 patient a une cardiopathie hypertrophique; une dysfonction du VG a été retrouvée chez 4 patients.

**Retentissement rénal:** 6 patients (17,6%) présentent une insuffisance rénale; 9 sujets (21,3%) présentent une protéinurie positive.

### Traitement de l'HTA

**Figure 5:**
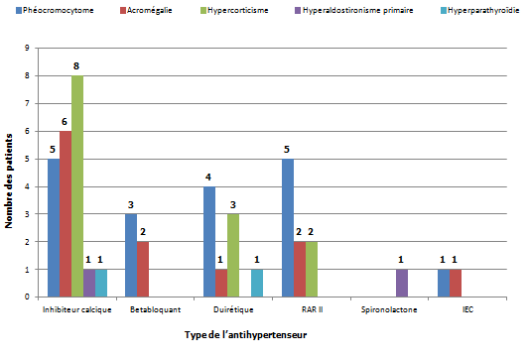
Prescription des antihypertenseurs en fonction de l’étiologie de l'HTA endocrine

**Prescription des antihypertenseurs en fonction de l’étiologie**: La classe thérapeutique prescrite est dominée par les inhibiteurs calciques (Figure 5).

**Répartition en fonction du nombre d'antihypertenseurs:** Dans 12 cas soit 26,6%, une bithérapie a été indiquée; la trithérapie a été prescrite chez 15 patients soit 33,3%; 5 patients ont nécessité la mise sous 4 antihypertenseurs, soit 8,9%; 14 patients ont été sous monothérapie soit 31,1%.

**Figure 6:**
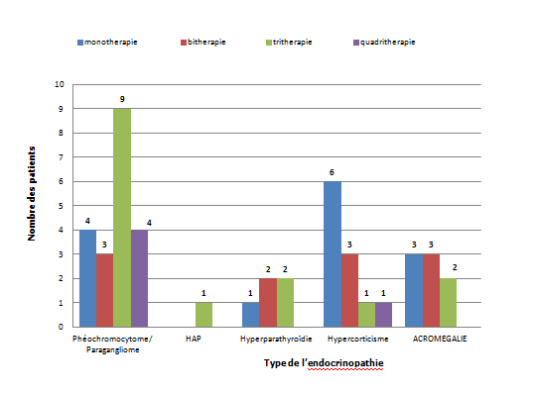
Nombre d'antihypertenseurs en fonction de l’étiologie

**Nombre d'antihypertenseurs en fonction des étiologies:** Le recours à la trithérapie était nécessaire dans 9 cas présentant un phéochromocytome et/ou paragangliome; le recours à 4 antihypertenseurs était indiqué chez 4 cas de phéochromocytome et dans 1 cas de syndrome de cushing (Figure 6).

**L’évolution après traitement étiologique:** Le traitement étiologique était possible dans 27 cas, parmi lesquels 16 patients ont normalisé leur TA après la chirurgie.

## Discussion

La prévalence de l'HTA secondaire a augmenté à partir des années 90, probablement du fait de l'amélioration des méthodes d'exploration, du dépistage hormonal et de l'imagerie [2, 8]. Elle n'est connue qu’à partir de séries hospitalières [4, 8, 9]. Elle est < 5% dans la population générale, elle atteint 13% chez les hypertendus hospitalisés dans un service hospitalo-universitaire et dépasse 30% chez les hypertendus résistant au traitement. Parmi ces HTA secondaires, les plus fréquentes et les plus fréquemment curables sont les HTA surrénales [4]. L'HTA n'est pas la manifestation dominante des hypercorticismes. En revanche, elle est quasi constante dans l'hyperaldostéronisme primaire (HAP) et dans les phéochromocytomes (PH) ou paragangliomes (PGL) [4]. Les HTA surrénales sont généralement révélées par les manifestations de l'hypersécrétion; excès d'aldostérone dans l'HAP, excès de catécholamines dans les PH/PGL. Parfois la tumeur est au premier plan: c'est la découverte fortuite d'une masse surrénale chez un hypertendu "un incidentalome" qui amène à la recherche d'une hypersécrétion et au diagnostic d'HTA surrénale.

### Les phéochromocytomes et paragangliomes

Le phéochromocytome est une tumeur neuroendocrine rare dont la prévalence généralement estimée à partir de séries hospitalières chez des patients hypertendus est de l'ordre de 1-2 sur 10 000. Dans les séries autoptiques, sa prévalence est de 0,3 à 0,95% [5, 10, 11]. L'HTA en est le signe cardinal mais n'est pas constante. Elle est particulièrement évocatrice lorsqu'elle est associée à la triade clinique: céphalées, sueurs et palpitations. La sensibilité et la spécificité de cette triade chez un patient hypertendu sont respectivement de 90,9 et de 93,8% [12, 13]. L'HTA dans les phéochromocytomes peut être paroxystique dans 48%, permanente dans 29%. Une absence d'HTA peut être notée chez 13% des phéochromocytomes [5]. L'HTA est permanente ou paroxystique du fait d'une sécrétion irrégulière des catécholamines par la tumeur. Les paroxysmes peuvent apparaître spontanément ou lors de circonstances déclenchantes (exemple: antefléxion, miction, défécation, etc...). L'HTA permanente est habituellement associée à la sécrétion de norépinéphrine. Une hypotension paradoxale est décrite surtout en cas de sécrétion d’épinéphrine ou de dopamine, mais il existe des cas rapportés où l'hypotension est associée à une hypersécrétion de noradrénaline [14]. Des formes à pression artérielle normale sont possibles, la vasoconstriction étant masquée par une hypovolémie. Les manifestations cardiaques du phéochromocytome sont la conséquence soit des répercussions de l'HTA, soit de l'imprégnation catécholaminergique prolongée. Elles peuvent être révélatrices ou compliquer l’évolution d'un phéochromocytome symptomatique méconnu [12, 15]. L'HTA est guérie dans 75% des cas mais le risque de récidive doit être expliqué au patient. Normalisation de la TA et trouble du rythme après 3 jours de la chirurgie [15, 16]. Dans notre série, le phéochromocytome est la cause la plus fréquente d'HTA endocrine avec un pourcentage de 38% des cas. Il s'agit dans la majorité des cas d'une HTA sévère grade III.

### L’ Hypercorticisme

Le syndrome de Cushing reste une cause rare d'HTA, sa prévalence dans des centres spécialisés en HTA entre 1975 et 2003 varie de 0,1 à 1% [17]. Des enquêtes rétrospectives ont rapporté que l'obésité faciotronculaire est fréquente chez les patients atteints d'un syndrome de Cushing et qu'il est difficile de les différencier des patients hypertendus compliqués d'obésité, en particulier chez les femmes [5]. L'HTA liée à l'hypercorticisme se caractérise par l'absence de diminution nocturne de la pression artérielle (PA). La prévalence de l'HTA chez les patients atteints d'un syndrome de Cushing endogène est de 80% (20% dans le syndrome de Cushing Iatrogène) [7, 18]. Les glucocorticoïdes sont impliqués de plusieurs façons dans la régulation de la PA. Le cortisol a une affinité pour le récepteur minéralocorticoïde identique à celle de l'aldostérone. En présence d'un hypercortisolisme, en revanche, la conversion du cortisol en cortisone peut être saturée, entraînant une stimulation du récepteur minéralocorticoïde, une majoration de la réabsorption sodée par les reins et donc une élévation de la PA. Le cortisol élève également la PA par d'autres mécanismes: la vasoconstriction artérielle et une action sur le système nerveux sympathique [14, 17]. Dans notre série, le syndrome de cushing représente 24% des étiologies de l'HTA endocrine.

### L'Hyperaldosteronisme primaire

L'HAP est une hypersécrétion autonome d'aldostérone, non adaptée et non stimulée par le système rénine-angiotensine. Les causes dominantes sont l'hyperplasie idiopathique et l'adénome produisant de l'aldostérone (APA) qui représentent 60 et 30% des cas respectivement [2, 4]. L'HAP est la cause la plus fréquente de l'HTA secondaire avec une prévalence estimée à 5- 13% des patients hypertendus, soit près de 3% de la population [8, 19]. La prévalence de l'HAP est encore plus élevée chez les patients ayant une HTA de grade 3 ou résistante au traitement (20%) (14), elle était évaluée entre 0,5% et 2% de la population hypertendue. Cependant, ce taux a connu une augmentation avec la standardisation des méthodes de dépistage. Les arguments sont suffisamment nombreux dans la littérature pour convaincre de l'intérêt du diagnostic et de la prise en charge de l'HAP [5, 14]. Les patients porteurs d'un adénome de Conn présentent aussi des caractéristiques hémodynamiques particulières, comparativement aux hypertensions essentielles avec une augmentation de l'index systolique et non significative de l'index cardiaque [20, 21]. Ces différents facteurs peuvent expliquer, au moins en partie, la plus grande prévalence des AVC hémorragiques et de dissection aortique constatés dans les HAP par rapport aux HTA essentielles [22]. Même si le taux de succès opératoires dans les adénomes latéralisés est imparfait, puisque entraînant la guérison dans 35 à 50% des cas, améliorant le niveau tensionnel dans 2/3 à 3/4 des cas, un bénéfice cardiovasculaire global est probable [22]. L'HTA peut persister chez 33à 77% des malades opérés pour adénome de Conn [6]. Parmi les facteurs prédictifs de persistance d'une HTA: l’âge avancé, la durée d’évolution de l'HTA et l'absence de réponse à un traitement d’épreuve par spironolactone en préopératoire. Chez la quasi-totalité des patients chez qui un traitement antihypertenseurs reste nécessaire en postopératoire, la chirurgie permet de diminuer le nombre de médications anti HTA et de guérir l'hypokaliémie [6]. Dans notre série, nous avons un patient présentant un HAP, ceci peut être expliqué par la taille faible de l’échantillon qui peut être non représentative et le fait qu'on n'est pas un centre référence d'HTA.

### L'acromégalie

L'hypersécrétion chronique d'hormone de croissance, est à l'origine d'anomalie cardiaque et vasculaire, aboutissant à une HTA. Plusieurs facteurs sont à l'origine de cette HTA: une hypervolémie responsable d'une augmentation du débit cardiaque, une dysfonction diastolique secondaire aux effets de l'hormone de croissance sur les cardiomyocytes, et un remodelage vasculaire responsable d'une augmentation des résistances périphériques [23]. La pathologie cardiovasculaire représente une cause importante de décès dans l'acromégalie; au moment du diagnostic, 60% des patients présentent une arythmie cardiaque, une HTA ou une valvulopathie. La prévalence de l'HTA dans l'acromégalie varie de 18 à 60% dans les différentes séries. Son incidence est plus élevée que dans la population générale [23]. Le traitement de l'acromégalie ne permet pas de guérir l'HTA chez la majorité des patients [9, 21]. Dans notre série, nous avons noté un pourcentage de 18% d'acromégalie chez les patients présentant une HTA endocrine

### Hyperparathyroïdie

L'hypercalcémie de l'hyperparathyroïdie primaire est associée à une élévation des chiffres tensionnels. Plusieurs hypothèses ont été proposées: l'hyperparathyroïdie entraîne une hypercalcémie qui altère les propriétés vasodilatatrices des cellules endothéliales [24]. Dans les situations d'hypercalcémie, il existe une plus grande concentration de noradrénaline et une réponse plus importante à la noradrénaline exogène. La prévalence de l'HTA est plus fréquente chez les patients qui ont une hyperparathyroïdie que dans la population générale, elle est de 30% dans l'HTA associée à l'hyperparathyroïdie peut également être survenir comme une complication de l′hypercalcémie induite par l'insuffisance rénale et dans le cadre d'une néoplasie endocrinienne multiple de type 2 (en association à un phéochromocytome) [17, 24]. L'existence d'un bénéfice tensionnel après traitement chirurgical de l'hyperparathyroïdie est controversée [14]. Dans notre série, nous avons noté un pourcentage de 11,11% l'hyperparathyroïdie primaire chez les patients présentant une HTA endocrine.

## Conclusion

Les hypertensions endocrines sont souvent d'origine surrénalienne et constituent les causes curables d'HTA. Leur diagnostic reste difficile du fait de l'absence de spécificité clinique et des difficultés d'interprétation des dosages hormonaux. Il n'est pas exceptionnel que l'HTA soit l'unique manifestation d'une maladie surrénale (notamment dans l'HAP). Ces pathologies nécessitent une prise en charge spécialisée et le plus souvent multidisciplinaire. Cependant, il existe une possibilité fréquente de guérison, à l'inverse de l'HTA essentielle. Ces pathologies nécessitent une prise en charge spécialisée et le plus souvent multidisciplinaire. Il est donc très important de dépister une hypertension endocrine devant toute HTA sévère, résistante au traitement chez un jeune, ou en présence de signes cliniques, biologiques ou radiologiques évocateurs. Nous avons noté dans cette étude le caractère sévère et résistant de l'HTA. Les étiologies étaient dominées par le phéochromocytome. Néanmoins les limites de cette étude peuvent être résumées dans la faible taille de l’échantillon et le fait qu'on n'est pas un centre référence d'HTA.

### Etat des connaissances sur le sujet

- HTA endocrine est une pathologie rare d'HTA mais curable;
- HTA d'origine endocrine peut engager le pronostic vital;
- Savoir penser à cette étiologie d'HTA devant telles endocrinopathies.

### Contribution de notre étude à la connaissance

- Prévalence très probablement sous estimée de l'HTA endocrine dans les pays en voie de développement;
- Nécessité de réaliser des centres de référence pour cette grave pathologie dans nos pays.
